# Appropriate Sampling and Longer Follow-Up Are Required to Rigorously Evaluate Longevity of Humoral Memory After Vaccination

**DOI:** 10.4049/immunohorizons.2300057

**Published:** 2024-06-10

**Authors:** Vitaly V. Ganusov

**Affiliations:** Host-Pathogen Interactions Program, Texas Biomedical Research Institute, San Antonio; Department of Microbiology, University of Tennessee, Knoxville, TN

## Abstract

One of the goals of vaccination is to induce long-lived immunity against the infection and/or disease. Many studies have followed the generation of humoral immunity to SARS-CoV-2 after vaccination; however, such studies typically varied by the duration of the follow-up and the number of time points at which immune response measurements were done. How these parameters (the number of time points and the overall duration of the follow-up) impact estimates of immunity longevity remain largely unknown. Several studies, including one by Arunachalam et al. (2023. *J. Clin. Invest.* 133: e167955), evaluated the humoral immune response in individuals receiving either a third or fourth dose of mRNA COVID-19 vaccine; by measuring Ab levels at three time points (prior to vaccination and at 1 and 6 mo), Arunachalam et al. found similar half-life times for serum Abs in the two groups and thus suggested that additional boosting is unnecessary to prolong immunity to SARS-CoV-2. I demonstrate that measuring Ab levels at these three time points and only for 6 mo does not allow one to accurately evaluate the long-term half-life of vaccine-induced Abs. By using the data from a cohort of blood donors followed for several years, I show that after revaccination with vaccinia virus, vaccinia virus–specific Abs decay biphasically, and even the late decay rate exceeds the true slow loss rate of humoral memory observed years prior to the boosting. Mathematical models of Ab response kinetics, parameterized using preliminary data, should be used for power analysis to determine the most appropriate timing and duration of sampling to rigorously determine the duration of humoral immunity after vaccination.

## Introduction

“To consult the statistician after an experiment is finished is often merely to ask him to conduct a post mortem examination. He can perhaps say what the experiment died of.”—Ronald Fisher

The Holy Grail for vaccinologists is to induce long-term effective immunity against infections, such as against SARS-CoV-2, the cause of the recent COVID-19 pandemic (1). It is well known that specific Abs to the spike protein of SARS-CoV-2 account for disease protection (2), and it is of great interest to discover how long such Abs persist postinfection or various forms and regimens of vaccinations (3, 4). In one such study, Arunachalam et al. (4) compared the durability of Ab levels in persons who received either three or four vaccinations with Pfizer or Moderna mRNA vaccines. Specifically, authors immunized previously vaccinated volunteers with mRNA vaccines and measured S-protein binding Abs or neutralizing Abs at three time points: prior to vaccination, at ∼1 mo, or at 6 mo after vaccination. Then Arunachalam et al. (4) averaged Ab titers for all volunteers and estimated the rate at which the titers declined between 1 and 6 mo postvaccination (specific measurement times varied somewhat between individuals). The authors found that the decay of Abs between 1 and 6 mo was similar in individuals receiving either third or fourth vaccinations and thus concluded that “[t]he durability of serum Ab responses improves only marginally following booster immunizations with the Pfizer-BioNTech or Moderna mRNA vaccines,” inferring that extra boosters are unnecessary to confer long-term immunity (4).

The duration of immunity after COVID-19 vaccination remains debated and is complicated by continuous evolution of SARS-CoV-2 to evade Ab response. The loss of neutralizing activity of serum of vaccinated individuals against novel SARS-CoV-2 variants such as Omicron is well documented (5, 6); however, other studies suggest that immunity to some variants may be long-lived (7). Unfortunately, the discussion around the duration of immunity against SARS-CoV-2 has typically used semiquantitative descriptors such as “short-lived” or “long-lived” without rigorous evaluation and quantification of the kinetics of Ab generation and loss and estimation of Ab half-life times. Furthermore, previous studies may have been underpowered with limited sampling and short duration to rigorously evaluate the true longevity of humoral memory to SARS-CoV-2 after mRNA vaccination. In particular, it was unclear if measuring Ab titers at 1 and 6 mo postvaccination, as was done by Arunachalam et al. (4), uses the proper time points to rigorously evaluate the longevity of humoral immunity.

In this study, we use mathematical modeling to provide estimates of the kinetics of loss of Abs specific to vaccinia virus (VV) and tetanus that are thought to have relatively long half-life times (8, 9). We show that to evaluate the true longevity of humoral memory, measurements of immune response need to include more time points early after the vaccination, around the expected peak of the immune response, and for several months after the immune response peak. We propose that to rigorously address questions on the kinetics and longevity of vaccine-induced immunity, the appropriate power analysis in consultation with mathematical modeling experts may be needed.

## Materials and Methods

### Experimental data

Experimental data on kinetics of Ab titers in plasma of blood donors have been provided by Drs. Mark Slifka and Ian Amanna and are from their previous publication (8). In this study, we specifically analyzed the data on the kinetics of VV- and tetanus-specific Abs from four subjects shown in Fig. 2 of Amanna et al. (8). We focused specifically on VV- and tetanus-specific Abs because revaccination with these Ags induced robust recall responses in all individuals. To fit mathematical models to the Ab kinetics after revaccination, we only included the first measurement before the peak of response and all the available data after revaccination. Data on the dynamics of Ab-secreting cells (ASCs) and Ab titers following boost immunization with VV (donor 97) were published previously (10). As far as we are aware, the listed donor identifiers are not known to anyone outside the research group that performed these measurements.

### Mathematical model for the kinetics of humoral immune response

In experiments involving immunization, it is common to only measure the Ab titers (either Ag-binding titers or neutralizing titers). To describe the kinetics of Ab response for those data, we adapt a simple *T*_on_*−T*_off_ model proposed to describe kinetics of the T cell response to viruses (10–12). In this model, immune response gets activated at *T*_on_ time and grows exponentially at a per capita rate ρ. The immune response peaks at time *T*_off_ and then decays at two rates, *δ*_1_ and *δ*_2_, that biologically correspond to ASCs with different longevity (given at frequency *f*_1_ = 1 *−* *f_m_* and *f*_2_ = *f_m_*, respectively):

$$A\left(t\right)\;=\;\{\begin{array}{ll} A_{0}, & \text{if}\;t<T_{ο\text{n},}\\ A_{0}{\text{e}}^{\rho \left(t-T_{\text{on}}\right)}, & \text{if}\;T_{\text{on}}\;\leq \;t<T_{\text{off},}\\ A_{0}{\text{e}}^{\rho \left(T_{\text{off}}-T_{\text{on}}\right)}\left(\left(1-f_{m}\right)e^{-\delta_{1}\left(t-T_{\text{off}}\right)}\;+\;f_{m}{\text{e}}^{-\delta_{2}\left(t-T_{\text{off}}\right)}\right), & \text{otherwise,} \end{array}$$ (1)

where *A*_0_ is the initial Ab titer (prior to boosting). Note that the half-life of humoral memory is determined by the lowest of the two decay rates *δ*_1_ and *δ*_2_, *T*_1/2_ = log_2_/min(*δ*_1_, *δ*_2_).

In some situations, experimental data do not include measurements of Ab titers during the expansion phase of the immune response after vaccination. In such cases, the data only include kinetics of Ab decline after a peak. To describe the kinetics of Ab decay after the peak following VV or tetanus revaccination, we used Eq. 1 with *t > T*_off_ to extend Ab dynamics driven by a different number *n* of subpopulations of ASCs with different lifespans:

$$\frac{\text{d}A}{\text{d}t}\;=\;\sum_{i=1}^{n}A_{0i}{\text{e}}^{-\delta_{i}t},$$ (2)

where *A* is the Ab titer from all subpopulations, *A*_0_*_i_* is the size of the *i*^th^ subpopulation, and *δ_i_* is the decay rate of the *i*^th^ subpopulation of ASCs. The ultimate half-life of Abs is determined by the slowest decay rate *δ* = min(*δ_i_*) with the corresponding half-life time *T*_1/2_ = ln(2)/*δ*. To determine how many subpopulations are needed to best describe the kinetics of Ab loss after the peak, we vary *n =* 1 … 3 and compare the quality of the model fits using the *F*-test for nested models (13).

In other situations, the dynamics of both ASCs and Abs are followed over time (10). To describe the dynamics of both ASC and Ab titer response following revaccination, we use a model proposed previously (10). In the model, ASC number (*P*) expands exponentially after a delay *T*_on_, peaks at *T*_off_, and then some ASCs die and some convert to a long-lived plasma cell phenotype at a rate *δ_A_*. At *T*_mem_, long-lived plasma cells are formed; they decay at rate *δ_M_*. ASCs produce Abs at a rate *p*, and Abs decay at a rate *δ_a_*:

$$\frac{dP}{dt}\;=\;\{\begin{array}{ll} 0, & \text{if}\;t\;<\;T_{\text{on}},\\ \rho P, & \text{if}\;T_{\text{on}}\;\leq \;t\;<\;T_{\text{off}},\\ -\delta_{A}P, & \text{if}\;T_{\text{off}}\;\leq \;t\;<\;T_{\text{mem}},\\ -\delta_{M}P, & \text{otherwise}, \end{array}$$ (3)

$$\frac{dA}{dt}\;=\;pP\;-\;\delta_{a}A,$$ (4)

where *p* is the rate of Ab production by a virus-specific plasma cell (or more precisely, per 1% of ASCs in the blood). Also, *δ_a_* is the natural decay rate of Abs assumed to be *δ_a_* = 0.0495 d*^−^*^1^, corresponding to a half-life of IgG of 2 wk (14, 15). In our example, *δ_M_* = 0 because this provided the best fit of the model to data (10).

### Statistics

The models were fitted to data using nonlinear least squares by logarithmically transforming the model predictions and the data. We used routine FindMinimum in Mathematica for nonlinear least squares. Nested alternative models were compared using the likelihood ratio test (also known as the *F*-test for nested models), and all models were compared using Akaike information criterion (AIC) for small sample sizes (13, 16). The model fit with the lowest AIC value is considered the best among tested models. An AIC difference *<*2 between two models indicates that the two models fit the data with similar quality, and a difference in AIC values *>*4 is considered statistically large to indicate that the model with smaller AIC fits the data statistically better.

## Results

From multiple studies in mice, monkeys, and humans, it is clear that the kinetics of Ab decay after infection or vaccination are generally more complex than a simple exponentially decaying function would suggest (8, 10, 17–19). Specifically, after the peak immune response, Ab titers initially decline rapidly and approach their long-term decay kinetics over months. This, in part, is driven by the heterogeneity of populations of ASCs with different lifespans (Supplemental Fig. 1A, 1B). However, some studies still use a simple, exponentially declining function to describe the kinetics of Abs after vaccination. Specifically, Arunachalam et al. (4) measured S-protein binding or neutralizing Abs after either a third or fourth immunization with mRNA-based COVID-19 vaccines and found that the rate of Ab decay from the peak at 1 mo after immunization did not differ between the two groups of vaccinees. The authors then concluded that a fourth immunization with mRNA COVID-19 vaccines does not improve the longevity of immune memory.

**FIGURE 1.**
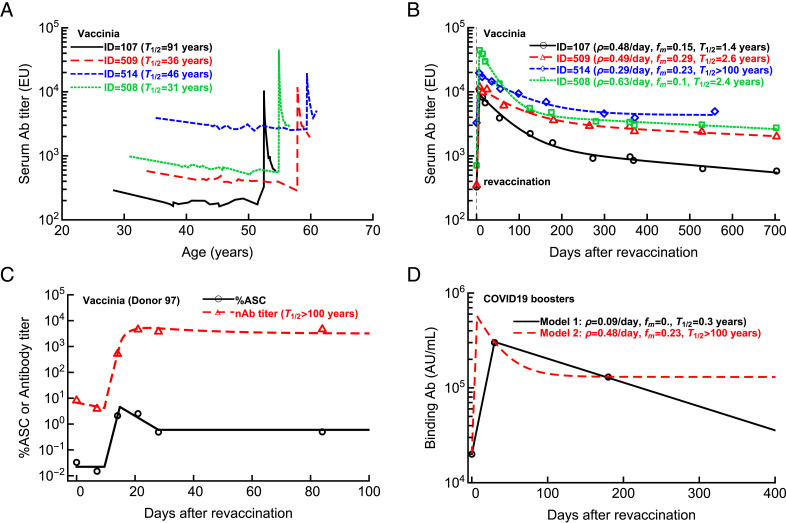
Biphasic decline of VV-specific Abs after revaccination. (**A**) We analyzed the kinetics of Ab titers in four long-term blood donors from subjects 1–4 shown in Fig. 2 of Amanna et al. (8). These individuals were followed up for 20+ years during which they had been revaccinated with VV. (**B**) We fitted a mathematical model of humoral immune response (Eq. 1) to subsets of the data that include revaccination and estimated the rate of Ab expansion (ρ), the proportion of Ab conversion into a long-lived population (*f_m_*), and the half-life of the humoral immunity (*T*_1/2_, B and see Table I for estimated model parameters). Supplemental Fig. 1C–1F shows dynamics of individual subpopulations of ASCs predicted by the model. (**C**) Kinetics of Ab response following VV revaccination in one volunteer (donor 97) suggests infinite half-life of the long-term memory [see main text for best fit parameters; these data were published previously (10)]. In (B) and (C), markers denote the data, and lines are predictions of the mathematical model. (**D**) Sparse measurements of Ab titers after vaccination allow alternative mathematical models (Eq. 1 with different parameter sets) with drastically different predicted longevities of Abs. Here, the markers are average Ab titers from Fig. 1Ciii of Arunachalam et al. (4), and lines are predictions of two alternative mathematical models with different assumed subpopulations of ASCs.

**FIGURE 2.**
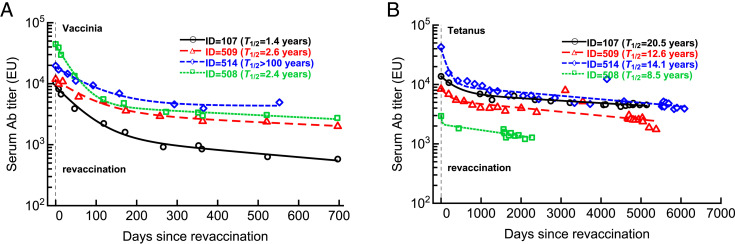
The approach to stable Ab levels takes months to years after revaccination with VV or tetanus vaccine. We fitted several alternative mathematical models for Ab decay (Eq. 2) to data by varying the number of subpopulations for four volunteers revaccinated with VV (**A**) or tetanus (**B**) vaccines. In the analysis, we included only the data from the peak Ab response at the first instance after an apparent revaccination (noted by the horizontal dashed line). In both cases, the models with two subpopulations fitted the data better than the model with a single subpopulation, but a larger model (*n =* 3) did not improve the model fit quality but did provide longer half-life times for persisting Abs. For each fit, we provide the estimated half-life time (*T*_1/2_) based on the slowest decay rate. In both panels, markers denote the data, and lines are predictions of the mathematical model. Parameters of the best fit models are shown in Table II. Note the different scales of the follow-up for boosting with two different vaccines. EU, ELISA units.

To investigate how the dynamics of Ab titers after revaccination relates to their long-term maintenance, we reanalyzed unique data from a cohort of long-term blood donors in which Ab titers to multiple vaccines were recorded over a long time period (8). For this analysis, we selected four individuals illustrated in Fig. 2 of Amanna et al. (8) who were followed for 20+ years and subsequently were revaccinated with VV (Figs. 1 and 2). The kinetics of Ab decay prior to revaccination showed remarkable stability of VV-specific Abs with half-life times of 31–91 y (Fig. 1A).

**Table I.** Estimates of parameters of Ab expansion and decay kinetics during revaccination with VV

Identifier	Vaccine	*A*_0_	ρ, 1/d	*T*_off_, d	*f_m_*	δ_1_, 1/d	δ_2_, 1/d
107	VV	327.2	0.48	6.94	0.15	0.018	0.00133
509	VV	348.8	0.49	7.09	0.29	0.014	0.00072
514	VV	3284.4	0.29	6.07	0.23	0.012	0
508	VV	710.7	0.63	6.5	0.10	0.029	0.00078

We fitted mathematical models (Eq. 1) to the data on Ab expansion and decay kinetics in four volunteers following revaccination with VV (Fig. 1B). In the model, *A*_0_ denotes the initial Ab titer, *ρ* is the rate expansion of ASCs, *T*_on_ = 0 is the time of revaccination, *T*_off_ is the time of the peak of the immune response, δ_1_ and δ_2_ are the rate of decay of the short-lived and longer-lived subpopulations of ASCs, respectively, and *f_m_* is the proportion of longer-lived ASCs in the overall population. Note that longevity of Ab response is best characterized by half-life time *T*_1/2_ = ln/δ_2_.

**Table II.** Estimates of parameters of Ab decay mathematical model fitted to data on Ab kinetics in four volunteers vaccinated with VV or tetanus vaccine

Identifier	Vaccine	*A*01	*A*02	*A*03	δ_1_, 1/d	δ_2_, 1/d	δ_3_, 1/d	*T*_1/2_, y
107	VV	7619.0	1387.9	0	0.0181	0.0013	0	1.45
509	VV	8123.8	3340.9	0	0.0138	0.0007	0	2.68
514	VV	14365.2	4335.8	0	0.0121	0	0	*>*100
508	VV	39419.9	4502.3	0	0.0293	0.0008	0	2.47
107	Tetanus	7038.6	6675.2	0	0.00009	0.00285	0	20.83
509	Tetanus	3096.0	5444.1	0	0.00524	0.00015	0	12.82
514	Tetanus	30789.1	9646.3	0	0.00689	0.00013	0	14.32
508	Tetanus	816.4	2127.5	0	0.04081	0.00022	0	8.66

We fitted several mathematical models (Eq. 2) to the data on Ab decay in four volunteers following revaccination with VV or tetanus vaccine (Fig. 2). Here, *A*_0_*_i_* denotes the Ab titer coming from *i*^th^ subpopulation of ASCs, and *δ_i_* is the rate of decay of the *i*th subpopulation over time.

We fitted a modification of the mathematical model of Ab response reported previously (10) to the data on Ab expansion and contraction after revaccination (Eq. 1; see Materials and Methods). This model includes the expansion of ASCs and the decay and formation of a long-lived subpopulation of plasma cells. Importantly, this model fitted the data extremely well (Fig. 1B) by assuming two subpopulations of ASCs; these data are clearly inconsistent with one ASC population model predicting an exponential decline after the peak (Supplemental Fig. 1). Two subpopulations of ASCs could be due to plasma cells generated in germinal centers (GCs) or extrafollicularly (i.e., outside of GCs) but could also arise due to different lifespans of GC-derived plasma cells (20).

Interestingly, we found much shorter half-life times of VV-specific Abs *T*_1/2_ = 1.4 *−* 2.6 y as compared with the half-life times prior to revaccination (except for volunteer 514, showing no long-term decay after revaccination; Fig. 1B). Given that these individuals were followed for nearly 2 y after VV revaccination, their VV-specific Abs levels still decayed more rapidly than during years prior to boosting. This analysis indicates that long follow-up studies are required before any conclusions can be drawn about the true longevity of vaccine-induced Abs.

It is not always possible to capture Ab dynamics shortly after vaccination, such as around the peak of immune response (8). An alternative approach to Eq. 1 to quantify the kinetics of Ab loss after vaccination is to ignore the expansion phase of the response and only focus on Ab decay. We therefore simplified our basic mathematical model to only include decay of Abs due to the presence of subpopulations of ASCs with different lifespans (Eq. 2), and we fitted the model to the data on the long-term VV- and tetanus-specific Abs dynamics (Fig. 2). Importantly, Ab decays were not well described by a simple exponential decay function, indicating that different subpopulations of ASCs with discordant lifespans may be present (Supplemental Fig. 1 and Supplemental Table I). We typically found that a model with two subpopulations fitted the data better than a model with a single, exponentially decaying population, but increasing the number of subpopulations to three did not improve the model fit (*p > *0.05 in likelihood ratio tests, and see AIC values in Supplemental Table I). The long-term loss of tetanus-specific Abs occurred at a slower rate than the loss of VV-specific Abs, which may be surprising because VV is a live vaccine and tetanus vaccine is a protein-based vaccine. The difference here likely lies in the longer follow-up times of tetanus-specific Abs, allowing more rigorous estimates of their half-life times (Fig. 2). In volunteers 508 and 509, a model with a single population of ASCs described the data slightly better than that with two ASC populations, but this difference was not significant based on AIC values (Supplemental Table I).

In our recent work, we formulated mathematical models aimed to describe simultaneously kinetics of ASCs and Ab titers following vaccination of humans (Eqs. 3 and 4) (10). This model links the kinetics of ASCs and Ab response in the blood and could accurately describe the rapid rise and maintenance of VV-specific Abs over time. Importantly, this analysis showed that to capture Ab loss after the peak, one needs to measure Ab titers frequently around the peak of the immune response (Fig. 1C; VV-specific response for donor 97 in the model given in Eqs. 3 and 4 are *P*_0_ = 0.022, *T*_on_ = 9.5 d, ρ = 1.02 d, *T*_off_ = 14.79 d, *δ_A_* = 0.15/d, *T*_mem_ = 28 d, *A*_0_ = 6.7, *p* = 261.3/d) (10).

What happens if one does not measure immune response frequently enough, such as at only three sparse time points? Then multiple different models could be consistent with the data. To illustrate this point, we used the model that described well the Ab response to VV (Eq. 1) and selected parameters that would allow matching the average Ab titers measured at three time points (0, 1, and 6 mo) chosen in the report of Arunachalam et al. (4). Importantly, we could choose two extreme sets of parameters that predict either relatively short-lived Ab response with half-life *T*_1/2_ = 0.3 y (model parameters: *A*_0_ = 20,000, ρ = 0.091/d, *T*_off_ = 30 d, *f_m_* = 0, δ_1_ = 0.0058/d, δ_2_ = 0.13/d) or extremely long Ab response (*T*_1/2_ *>* 100 y, model parameters: *A*_0_ = 20,000, ρ = 0.48/d, *T*_off_ = 7 d, *f_m_* = 0.23, δ_1_ = 0.04/d, δ_2_ = 0.000013/d; Fig. 1D). These results further demonstrate that measuring Ab titers at only three sparsely spaced time points does not allow one to rigorously evaluate the duration of humoral memory following revaccination.

## Discussion

By using data from cohorts of individuals vaccinated with VV or tetanus, we confirmed that the decay of serum Abs is best described by a model with at least two subpopulations of plasma cells with different lifespans (Figs. 1 and 2), as has been observed in humans and monkeys (8, 19). Evaluating the frequency of the longer-lived subset and its decay half-life time requires frequent sampling around the expected peak of immune response and for months after the vaccination. We illustrated that measuring serum Abs only at three time points as was done by Arunachalam et al. (4) does not allow one to infer the longevity of immunological memory, and such measurements will inaccurately predict the duration of protection (Fig. 1D).

So, how can we rigorously evaluate the duration of humoral immunity to SARS-CoV-2 after vaccination? Obviously, following revaccinated individuals for longer times may provide some useful information, but inapparent boosting of immunity following reinfection from currently circulating SARS-CoV-2 cannot be excluded. A better approach would be to improve the experimental design by using mathematical modeling–assisted power analyses (based on Eqs. 3 and 4) that should indicate the most informative time points at which samples should be collected to provide the most reliable estimate of the parameter of immunity that is being advocated, such as the Ab half-life times (21, 22). Using mathematical models based on a single population of ASCs should be avoided because these models do not accurately describe long-term Ab dynamics (Fig. 1B and Fig. 2). Furthermore, the presence of plasma cells with different lifespans is expected, given the basic immunology of extrafollicular and GC phases of humoral immune response (23). Indeed, a recent study suggested that mRNA-based COVID-19 vaccines do provide relatively long-lived humoral immunity if one follows Ab kinetics over longer periods of time (24).

Following Ab response longitudinally in individual volunteers may also allow one to use a more powerful approach of mixed effect modeling that may help better define average Ab half-life time and its variability between individuals (25). Providing actual estimates of Ab half-life times and relating those other known estimates [e.g., in (8)] will be a more rigorous approach than to use semiquantitative terms such as “short-lived” or “long-lived.” Linking Ab generation and decay kinetics to the Ab levels that are protective against COVID-19 will be necessary to fully define the biologically relevant duration of protection afforded by humoral immunity. Ultimately, better collaborations between experimentalists and mathematical modelers may help researchers to design more reliable experiments that provide rigorous estimates of the longevity of humoral immunity afforded by vaccination and (to paraphrase Ronald Fisher) “not let postmortem analysis to identify reasons the experiment died of.”

## Supplementary Material

Supplemental Material (PDF)
