# Supplementary material for: Appropriate Sampling and Longer Follow-Up Are Required to Rigorously Evaluate Longevity of Humoral Memory After Vaccination
Source: Immunohorizons. 2024 Jun 10;8(6):397–403. doi: 10.4049/immunohorizons.2300057 (PMC11220738; doi:10.4049/immunohorizons.2300057)
Supplement: Supplemental Material (PDF) [file IH_2300057_Supplemental_1.pdf]

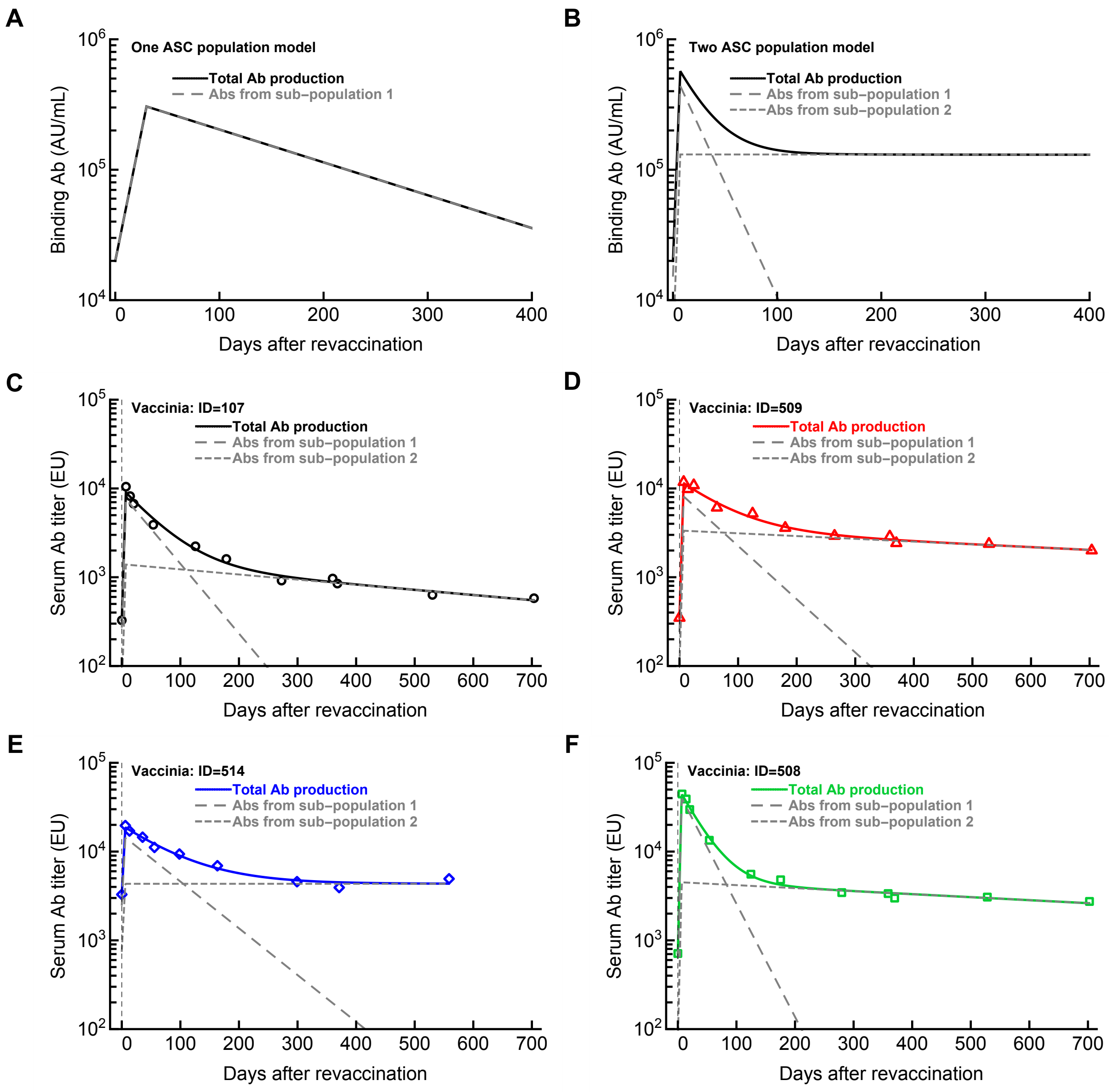

Supplemental Figure I: Differences in the dynamics of Ab response described by one or two population of ASCs. A-B: We plot predictions of the mathematical model (**eqn. (1)**) assuming that Ab production is driven by one (A) or two (B) populations of ASCs with different decay rates (see **Figure 1**D for model parameters). **C-D**: We show the data on Ab kinetics after VV revaccination and best fit of the model (**eqn. (1)**) that assumed two populations of ASCs. Markers denote the data, solid lines are predictions of the model, and dashed lines show the dynamics of each of the two populations of ASCs (see **Figure 1**B for model parameters).

**Vaccination**	**ID**	**# of populations**	**# of parameters**	**SSR**	**AIC**
Vaccinia	107	1	2	0.416	-26.58
Vaccinia	107	2	4	0.021	**-47.1**
Vaccinia	107	3	6	0.008	-28.32
Vaccinia	509	1	2	0.416	-26.58
Vaccinia	509	2	4	0.012	**-52.87**
Vaccinia	509	3	6	0.012	-23.53
Vaccinia	514	1	2	0.127	-27.55
Vaccinia	514	2	4	0.01	**-31.21**
Vaccinia	514	3	6	0.01	64.79
Vaccinia	508	1	2	0.764	-19.91
Vaccinia	508	2	4	0.01	**-55.21**
Vaccinia	508	3	6	0.004	-36.8
Tetanus	107	1	2	0.081	-129.44
Tetanus	107	2	4	0.016	**-162.35**
Tetanus	107	3	6	0.015	-156.4
Tetanus	509	1	2	0.269	**-111.72**
Tetanus	509	2	4	0.238	-109.04
Tetanus	509	3	6	0.238	-101.81
Tetanus	514	1	2	0.55	-103.05
Tetanus	514	2	4	0.196	**-126.23**
Tetanus	514	3	6	0.17	-123.34
Tetanus	508	1	2	0.023	**-89.18**
Tetanus	508	2	4	0.014	-87.86
Tetanus	508	3	6	NA	NA

Supplemental Table I: Two population model typically describes better the kinetics of Ab loss after VV or tetanus revacciation. We fitted mathematical models (**eqn. (2)**) to the data on Ab decay in four volunteers following revaccination with VV or tetanus vaccine (**Figure 2**) assuming one, two, or three populations of ASCs. We show the number of fitted parameters, the sum of squared residuals (**SSR**), and AIC. We could not fit the model with three populations to the data in volunteer 508 after tetanus revaccination (denoted as NA) because the data are well described by a single, exponentially decaying function. The lowest AIC value among three alternative models fitted to each dataset is highlighted in bold.
